# Comparison of the Bifidogenic Effects of Goat and Cow Milk-Based Infant Formulas to Human Breast Milk in an *in vitro* Gut Model for 3-Month-Old Infants

**DOI:** 10.3389/fnut.2020.608495

**Published:** 2020-12-11

**Authors:** Sophie Gallier, Pieter Van den Abbeele, Colin Prosser

**Affiliations:** ^1^Dairy Goat Co-operative (NZ) Ltd, Hamilton, New Zealand; ^2^ProDigest BVBA, Ghent, Belgium

**Keywords:** human milk, infant formula, goat milk, cow milk, gut microbiota, short-chain fatty acid (SCFA)

## Abstract

Human milk contains prebiotic components, such as human milk oligosaccharides (HMOs), which stimulate the growth of specific members of the infant gut microbiota (e.g., *Bifidobacteria*). Plant-based or synthetic oligosaccharides are often added to infant formulas to simulate the bifidogenic effect of HMOs. Cow milk, the most common source of protein in infant formula, and goat milk, used increasingly in the manufacture of infant formula, contain naturally-occurring prebiotics. This study compared the upper gastrointestinal digestion and subsequent colonic fermentation of human milk vs. goat and cow milk-based infant formulas (goat IF and cow IF, respectively), without additional oligosaccharides using an *in vitro* model for 3-month-old infants based on the Simulator of the Human Intestinal Microbial Ecosystem (SHIME®). First, a dialysis approach using 3.5 kDa membranes was demonstrated to simulate small intestinal absorption of carbohydrates in conditions similar to those *in vivo*. During the *in vitro* digestion experiment, oligosaccharides were detected in human milk and goat IF but barely detected in the cow IF. Further, all three milk matrices decreased colonic pH by boosting acetate, lactate, and propionate production, which related to increased abundances of acetate/lactate-producing *Bifidobacteriaceae* for human milk (+25.7%) and especially goat IF (33.8%) and cow IF (37.7%). Only cow IF stimulated butyrate production which correlated with an increase in *Lachnospiraceae* and *Clostridiaceae*. Finally, *Enterobacteriaceae* and *Acidaminococcaceae* also increased with all three milk matrices, while production of proteolytic metabolites (branched-chain fatty acids) was only detected for the cow IF. Overall, goat and cow milk-based formulas without added oligosaccharides impacted gut microbial activity and composition similarly to human milk. This suggests that even without supplementation of formula with oligosaccharides, whole goat milk, whole cow milk and cow milk ingredients already supply compounds in formulas that exert beneficial bifidogenic effects. Further clinical research is warranted to elucidate the effect of whole goat milk-based formulas on the infant gut microbiome.

## Introduction

Microbial communities inhabit the human bowel and carry out diverse and complex biochemical processing of compounds that escape digestion and absorption along the upper gastrointestinal tract (GIT). The human gut microbial communities are established just after birth and strongly affected by subsequent dietary patterns such as breastfeeding or formula feeding and introduction of solid food (1–3). Prebiotics are defined as “non-digestible food ingredients that beneficially affect the host by selectively stimulating the growth and/or activity of one or a limited number of bacterial species, already resident in the colon” (4). With respect to the infant diet, compounds with interesting prebiotic function are human milk oligosaccharides (HMOs). Due to their ability to resist acidic gastric conditions and enzymatic degradation in the upper GIT, they can exert their prebiotic effect in the lower GIT by specifically acting as nutrients and promoting the growth of *Bifidobacteria* and *Lactobacilli* in the colon (3, 5–7). *Bifidobacteria* are particularly well-adapted for the utilization of HMOs (8). A microbiota that is dominated by *Bifidobacteria* is considered protective, as it may activate the immune system and inhibit pathogens (9, 10). Some evidence suggests that *Bifidobacteria* and the production of short chain fatty acids (SCFAs) by gut bacteria may also protect against the development of allergy in infants (2, 3, 11).

Plant-based or synthetic oligosaccharides are often added to infant formulas to simulate the bifidogenic effect of HMOs. Infant formulas are commonly made from cow milk-derived ingredients (skim milk and whey protein powders), but goat milk is also a suitable milk source for formulas (12–14). Both goat milk and cow milk contain naturally-occurring oligosaccharides, albeit at lower concentrations and diversity than human milk (15, 16). However, compared to the profile of cow and sheep milk oligosaccharides, the profile of goat milk oligosaccharides is closer to that of human milk (15), with higher concentrations of fucosylated oligosaccharides and sialyloligosaccharides (16). Goat milk has also 4 and 10 times more oligosaccharides than cow milk and sheep milk, respectively (16). Goat milk has both acidic and neutral oligosaccharides, many of which are structurally comparable to HMOs (17–19). Therefore, the use of whole goat milk or specific goat milk fractions may provide some prebiotic benefits for the development of the maturing gut of the formula-fed infant.

Some studies have confirmed the potential of goat milk to influence the intestinal microbial community and metabolism in rodents (20, 21). Oligosaccharides isolated from goat milk promoted the growth of *Bifidobacteria* in *in vitro* models (22, 23). *Bifidobacteria* were also the most abundant microbes in stools of 2-month-old infants fed human milk, whole goat milk formula or whey-based cow milk formula (24). While there were no significant differences between the formula groups in the abundance of *Bifidobacteriaceae* or subspecies type, diversity analysis suggested that DNA sequences of microbiota were more similar when comparing the breast-fed and whole goat milk formula-fed infant groups than breast-fed and whey-based cow milk formula-fed infant groups (24). The aim of this study was to further investigate the observations made by Tannock et al. (24), by studying the effects of a whole goat milk-based and a whey-adjusted cow milk-based infant formula without any added prebiotics in comparison to human milk, using an *in vitro* model for 3-month-old babies, based on the Simulator of the Human Intestinal Microbial Ecosystem (SHIME®).

## Materials and Methods

### Chemicals and Test Products

All chemicals were obtained from Sigma-Aldrich (Overijse, Belgium) unless stated otherwise. Human milk was from a mother in the third month of lactation. Informed consent was obtained prior to milk collection and local ethics approval was granted by the Ethics Committee of Ghent University Hospital (Belgian registration number B670201523541). Further, two infant formulas were tested, one made from whole goat milk (goat IF) and one made from cow milk protein ingredients (cow IF) (Table 1). Infant formulas were manufactured by Dairy Goat Co-operative (N.Z.) Ltd (Hamilton, New Zealand) from pasteurized whole milk (goat or cow milk), skim milk powder (cow IF only), whey protein powder (cow IF only), lactose, vegetable oils (high oleic sunflower oil, coconut oil, canola oil, sunflower oil, soybean oil), minerals, marine fish oil (source of DHA), microbial oil (source of arachidonic acid), and vitamins. Neither formula had added oligosaccharides. Formulas were prepared by mixing 6.6 g of powder to 100 mL with water. This is half the concentration used when preparing a bottle feed but was required for the lactose absorption simulation experiment as described later, and therefore human milk was also diluted 1:1 with water.

**Table 1:** Macronutrient composition of the goat (goat IF) and cow (cow IF) milk-based infant formulas.

	**Units (per 100g powder)**	**Goat IF**	**Cow IF**
Energy	kJ	2,200	2,200
	kcal	510	510
Protein	g	10.1	10.1
Whey	%	20	63
Fat	g	26.7	26.7
Milk fat	g	13.1	8.2
Saturated	g	10.4	9.8
Mono-unsaturated	g	12.1	10.3
Poly-unsaturated	g	4.2	6.6
Carbohydrate	g	57.4	57.4
Lactose	g	57.4	57.4

### Experimental Design

**Figure 1:**
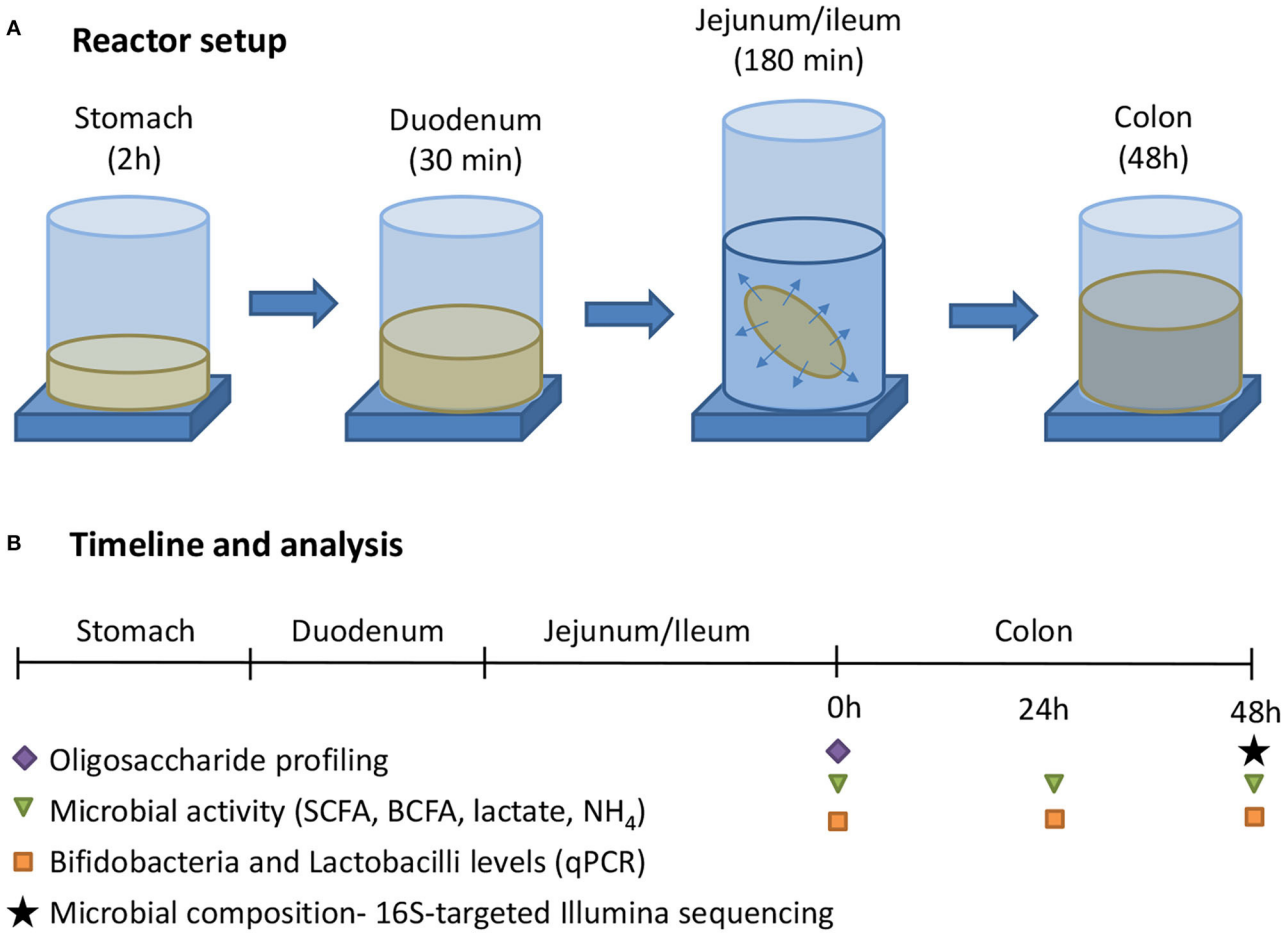
**(A)** The experimental procedure consisted of a sequential incubation of the milk matrices (or blank = water) in the simulated stomach, duodenum, jejunum/ileum (with simulation of small intestinal absorption via static dialysis), and colon. **(B)** Along the experiment, samples were collected for analysis of the oligosaccharide fractions, together with microbial activity, and composition.

The general reactor setup was adapted from the SHIME®, representing the GIT of the human adult as described by Molly et al. (25), to the digestive conditions of 3-month-old infants. Similar as how van den Abbeele et al. (26) adapted model parameters for human adult simulations, operational parameters for infants were adapted from the international consensus method of the INFOGEST consortium (27) that was recently extended for young infants (28). All three test products (cow IF, goat IF and human milk) were first subjected to upper gastrointestinal digestive and absorptive processes, after which they underwent colonic incubations (Figure 1). The treatment effects were compared to a blank consisting of water. All experiments were conducted in triplicate.

### Upper GIT Simulation of Milk Matrices

Except for the oral phase, which was omitted to simulate infant feeding condition, the residence times in the gastric and small intestinal incubations were similar to human adult conditions (26). Sixty milliliter of diluted milk matrices were mixed with simulated saliva medium (40 μL of solution containing 30 mg/mL α-amylase from porcine pancreas; 10% of the human adult level), pepsin (0.51 mL of a solution containing 20 mg of pepsin (R5015, Fiers, Zedelgem, Belgium; 18% of human adult level) per mL of 5 mM HCl), lipase (1.34 mL of a solution containing 50 mg/mL of Lipase from Rhizopus Oryzae; 100% of human adult level), lecithin (0.31 mL of a solution containing 13.5 mg/mL lecithin; 100% of human adult level), and gastric juice (2.3 mL of a solution containing 0.65 g KCl/L and 3.65 g NaCl/L). This mixture was incubated for 2 h at 37°C, during which a sigmoidal pH decrease was applied from 5.5 to 3.2 by adding 1M HCl. At the end of the incubation, the volume was increased to 75 mL with distilled water after which 10 mL was sampled. To the residual volume, 45 mL of a standardized enzyme and bile solution was added resulting in final amylase, lipase, chymotrypsin, bovine bile salts (BD Difco Oxgall, BD biosciences, Erembodegem, Belgium) and trypsin levels of 0%, 10, 50, 60, and 90% that of the human adult levels, respectively. Following a 30-min incubation at a pH of 6.5 and at 37°C, with stirring, a subsequent 3 h incubation at pH 6.7 was performed during which the intestinal content was incubated at the inside of a 3.5 kDa cut-off cellulose membrane, with the outside containing a dialysis solution (3.75 g/L NaHCO_3_). The ratio of intestinal content:dialysis solution was 1:2 and the dialysis solution was refreshed hourly to ensure a high diffusion gradient and maintain a driving force for dialysis. At the end of the small intestinal incubation, qualitative oligosaccharide profiles were determined in the intestinal content via HPAEC-PAD.

Three types of membranes (0.1–0.5 kDa, 0.5–1.0 kDa, and 3.5 kDa) using three types of compounds [lactose, lactalbumin, and fructo-oligosaccharides (FOS)] were tested (results not shown). The 3.5 kDa membrane led to the highest absorption rate of lactalbumin and lactose. However, a small fraction of FOS was absorbed with the 3.5 kDa membrane but not with the other two membranes. Hence, the 3.5 kDa membrane was chosen as the best compromise to minimize absorption of non-digestible carbohydrates and maximize removal of lactose and proteins to best reflect *in vivo* conditions. During a pre-test, the dialysis via a 3.5 kDa cut-off membrane, as described previously by van den Abbeele et al. (26), was validated by using two reference molecules (lactose and FOS), while using same volumes and refreshment procedures as outlined above.

### Oligosaccharide Profiling and Lactose Analysis via HPAEC-PAD

Concentrations of lactose during the pretest (to validate the dialysis membranes) were measured through high performance anion exchange chromatography with pulsed amperometric detection (HPAEC-PAD), using a ICS-3000 chromatograph (Dionex, Sunnyvale, CA, USA) equipped with a CarboPacPA20 column (Dionex). The mobile phase, at a flow rate of 0.5 mL/min, consisted of ultrapure water (eluent A) and 100 mM NaOH (eluent B). The following gradient was applied: 0 min, 95% A and 5% B; 20 min, 80% A and 20% B (linear change); 25 min, 80% A and 20% B; 26 min, 0% A and 100% B (linear change); 29 min, 0% A and 100% B; 30 min, 95% A and 5% B (linear change) and 32 min, 95% A and 5% B. Sample preparation involved initial dilution of the sample with ultrapure water followed by deproteinization with acetonitrile (1:1), centrifugation (24,400 × g, 10 min) and filtration (0.2 μm PTFE, 13 mm syringe filter, VWR International) prior to injection (5 μL) into the column. Calibration was performed using external standards.

Analysis of absorption and degradation of FOS (reference compound used during pretest to optimize absorption simulation), goat IF, cow IF and human milk during passage through the upper GIT was performed with HPAEC-PAD using a ICS-3000 chromatograph (Dionex) equipped with a CarbopacPA200 column (Dionex). The mobile phase, at a flow rate of 0.5 mL/min, consisted of ultrapure water (eluent A), 100 mM NaOH (eluent B), and 100 mM NaOH and 1M CH3COONa. The following gradient was applied: 0 min, 90%, 10% B, and 0% C; 7 min, 90%A, 10% B, and 0% C; 9 min, 50% A, 50% B, and 0% C (linear change); 25 min, 50% A, 50% B, and 0% C; 28 min, 41.75%, 56% B, and 2.25% C (linear change); 38 min, 41.75%, 56% B, and 2.25% C; 51 min, 6% A, 82% B, and 12%C (linear change); 61 min, 6% A, 74% B, and 20%C (linear change); 76 min, 6% A, 74% B, and 20%C; 85 min, 0% A, 60% B, and 40% C (linear change); 90 min, 90% A, 10% B, and 0% C (linear change); 95 min, 90% A, 10% B, and 0% C. Sample preparation involved initial dilution of the sample with ultrapure water followed by deproteinization with acetonitrile (1:1), centrifugation (24,400 × g, 10 min) and filtration (0.2 μm PTFE, 13 mm syringe filter, VWR International) prior to injection (5 μL) into the column. Qualitative fingerprints were generated by plotting the elution time (in min) against the detected signal (in nC).

### Colonic Incubation of Milk Matrices

After dialysis the intestinal content was subjected to a simulated colonic incubation as described by Marsaux et al. (29). Briefly, 49.5 mL colonic background medium [K_2_HPO_4_ 4.8 g/L; KH_2_PO_4_ 14.9 g/L; NaHCO_3_ 2.0 g/L; yeast extract 2.0 g/L; peptone 2.0 g/L; mucin 1.0 g/L; cysteine 0.5 g/L; polyoxyethylene (20) sorbitan monooleate 2.0 mL/L] was added to reactors, already containing 20 mL of upper GIT suspension. The reactors were sealed with rubber stoppers and rendered anaerobic by flushing with N_2_, after which 0.5 mL of a fecal inoculum was added. Stool samples of 3-month-old infants were collected according to the ethics approval from the Ethics Committee of Ghent University Hospital (Belgian registration number B670201523541). The stool inoculum from three 3-month-old infants were pre-screened with galactooligosaccharide and FOS (ratio 9:1) to select a representative inoculum. The selected stool inoculum from one donor showed a microbial profile that was very similar to that of young infants (i.e., high *Bifidobacterium* levels and high production of acetate and lactate) whereas the stool inocula from the other two donors were more similar to that of adults. The inoculum was prepared by suspending a freshly collected fecal sample at 7.5% (w/v) in anaerobic phosphate buffer (K_2_HPO_4_ 8.8 g/L; KH_2_PO_4_ 6.8 g/L; sodium thioglycolate 0.1 g/L; sodium dithionite 0.015 g/L). Throughout the 48 h incubation (at 37°C; Figure 1), samples were collected at 0, 24, and 48 h for microbial metabolic activity analysis [pH, gas, SCFA, lactate, and branched-chain fatty acid (BCFA) production] and *Bifidobacterium* quantification (via qPCR). At the final time point (48 h), in-depth community analysis via 16S-targeted Illumina sequencing was performed.

### Microbial Activity

Metabolic activity analysis at 0, 24, and 48 h included measurement of pH [measured via Senseline pH meter F410 (ProSense, Oosterhout, The Netherlands)] and gas production [measured via a pressure meter (hand-held pressure indicator CPH6200; Wika, Echt, The Netherlands)]. Further, total SCFA production was determined as the sum of acetate, propionate, butyrate and BCFAs (sum of isobutyrate, isovalerate, and isocaproate). The quantification method has previously been described by de Weirdt et al. (30). Ammonium determination was performed via a steam-distillation method followed by a titration as reported by de Boever et al. (31). Lactate was measured using a D-lactate/L-lactate kit (R-Biopharm, Mannheim, Germany).

### Microbial Composition

Samples from the colonic incubation were collected at 0, 24, and 48 h for assessment of microbial composition. Abundance of *Lactobacillus* and *Bifidobacterium* were quantified via qPCR. DNA was isolated from 1 mL of samples as described before by Boon et al. (32), with modifications as described by Duysburgh et al. (33). Subsequently, qPCR was performed on a QuantStudio 5 Real-Time PCR system (Applied Biosystems, Foster City, CA, United States). Each sample was run in technical triplicate and outliers with more than 1 CT difference were omitted. The qPCR for *Bifidobacteria* was performed as described previously by Rinttilä et al. (34), with the Bif243F (5′-TCGCGTCYGGTGTGAAAG-3′) and the Bif243R (5′-CCACATCCAGCRTCCAC-3′) primers, while *Lactobacilli* were quantified according to Furet et al. (35) with Llac05-F (5′-AGCAGTAGGGAATCTTCGGCA-3′) and Llac02-R (5′-GGGTAGTTACCGTCACTTGATGAG-3′) primers. Results are reported as log(16S rRNA gene copies/mL).

To get insight into the changes in relative abundance of the different microbial groups at overall community level, samples collected at 48 h were assessed via 16S-targeted Illumina sequencing (LGC genomics GmbH, Berlin, Germany) as described by Van den Abbeele et al. (36). Briefly, results obtained from the Illumina Miseq platform with v3 chemistry were presented as proportional values vs. the total amount of sequences within each sample and combined at family level. The reciprocal Simpson Diversity index was calculated as a measure of bacterial diversity, both in terms of species richness and evenness (37). The data used to create the OTU table that was used as a basis for the microbial community analysis in this paper have been deposited in the National Center for Biotechnology Information (NCBI) database (PRJNA675453).

### Statistics

Incubations were run in triplicate. All data analyses were conducted using SAS 9.4. Data on production of gas, pH and ammonium, and *Bifidobacteria* abundance (log10 transformed) were analyzed using repeated measures ANOVA followed by Tukey *post-hoc* test. Lactate and SCFA data were analyzed using one-way ANOVA followed by Tukey *post-hoc* test. A *P* ≤ 0.05 was considered to be significant. All values are reported as means ± standard deviation (SD).

## Results

### Optimization of Simulation of Small Intestinal Absorption

**Figure 2:**
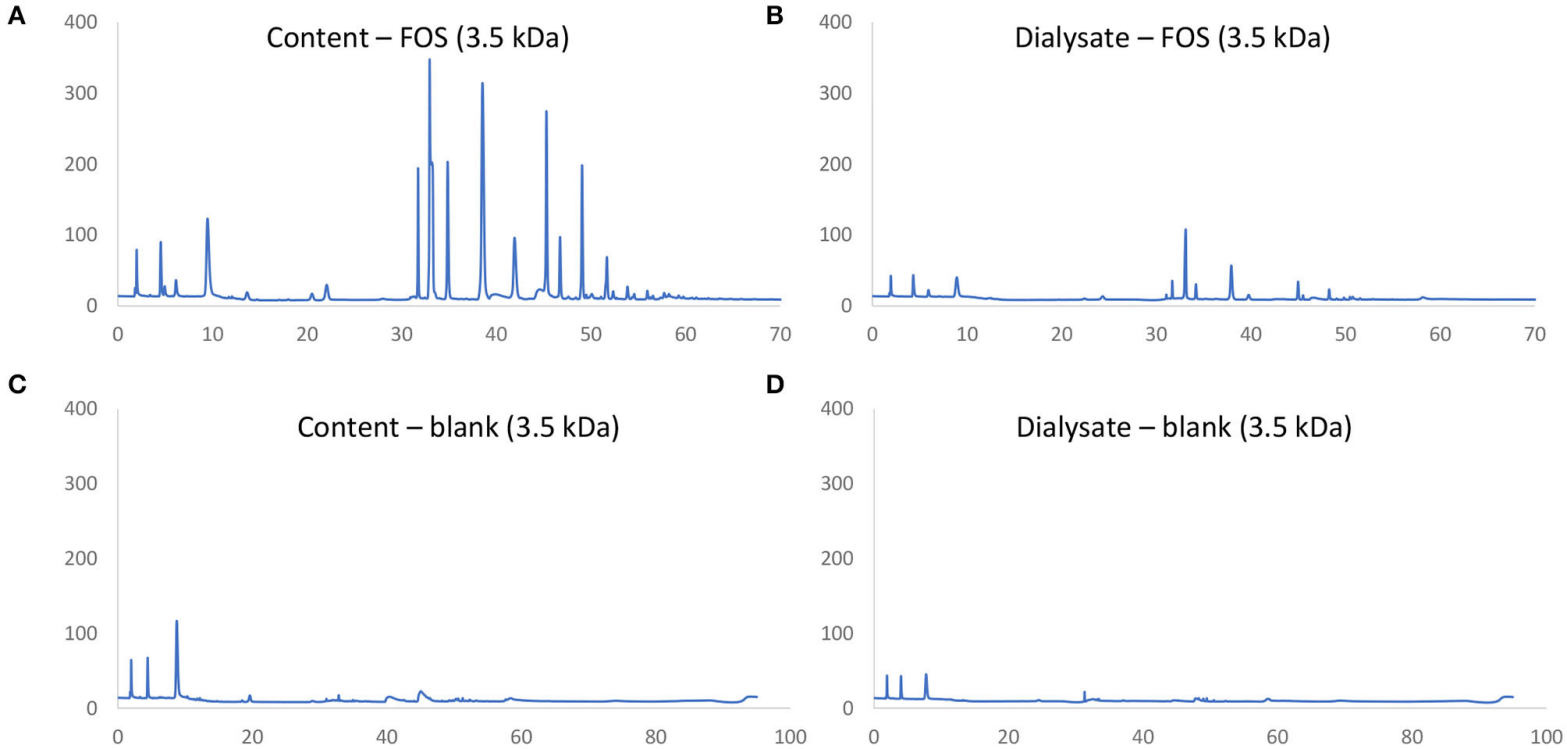
Chromatographic fingerprint of signal (nC) in function of time (min) obtained through HPAEC-PAD analysis of samples in the simulated intestinal content **(A,C)** or absorbed fraction **(B,D)** upon upper GIT transit of FOS **(A,B)** or a blank **(C,D)** using specific 3.5 kDa dialysis membranes to simulate small intestinal absorption.

First a novel dialysis approach was validated (using 3.5 kDa membranes) that would retain indigestible oligosaccharides at the inside of the membrane (simulated intestinal content), while allowing for diffusion (simulated absorption) of small mono- and disaccharides that are normally absorbed *in vivo* after digestion. FOS and lactose were used as reference substrates for the former and latter, respectively. First, upon comparison of chromatograms obtained via HPAEC-PAD for FOS (Figures 2A,B) vs. respective blanks (Figures 2C,D), it followed that the oligosaccharide fraction mainly eluted between 30 and 60 min, while the blank contained background peaks between 0 and 10 min. As only minor peaks appeared between 30 and 60 min in the chromatograms of samples of the dialysate upon applying the 3.5 kDa membrane (Figure 2B), the membrane was able to retain part of the oligosaccharide fractions of FOS. Further, dialysis with the 3.5 kDa membrane removed 52.2% of the lactose (data not shown). Therefore, the 3.5 kDa membrane was used for subsequent upper GIT simulations.

### Small Intestinal Incubations With Milk Matrices

**Figure 3:**
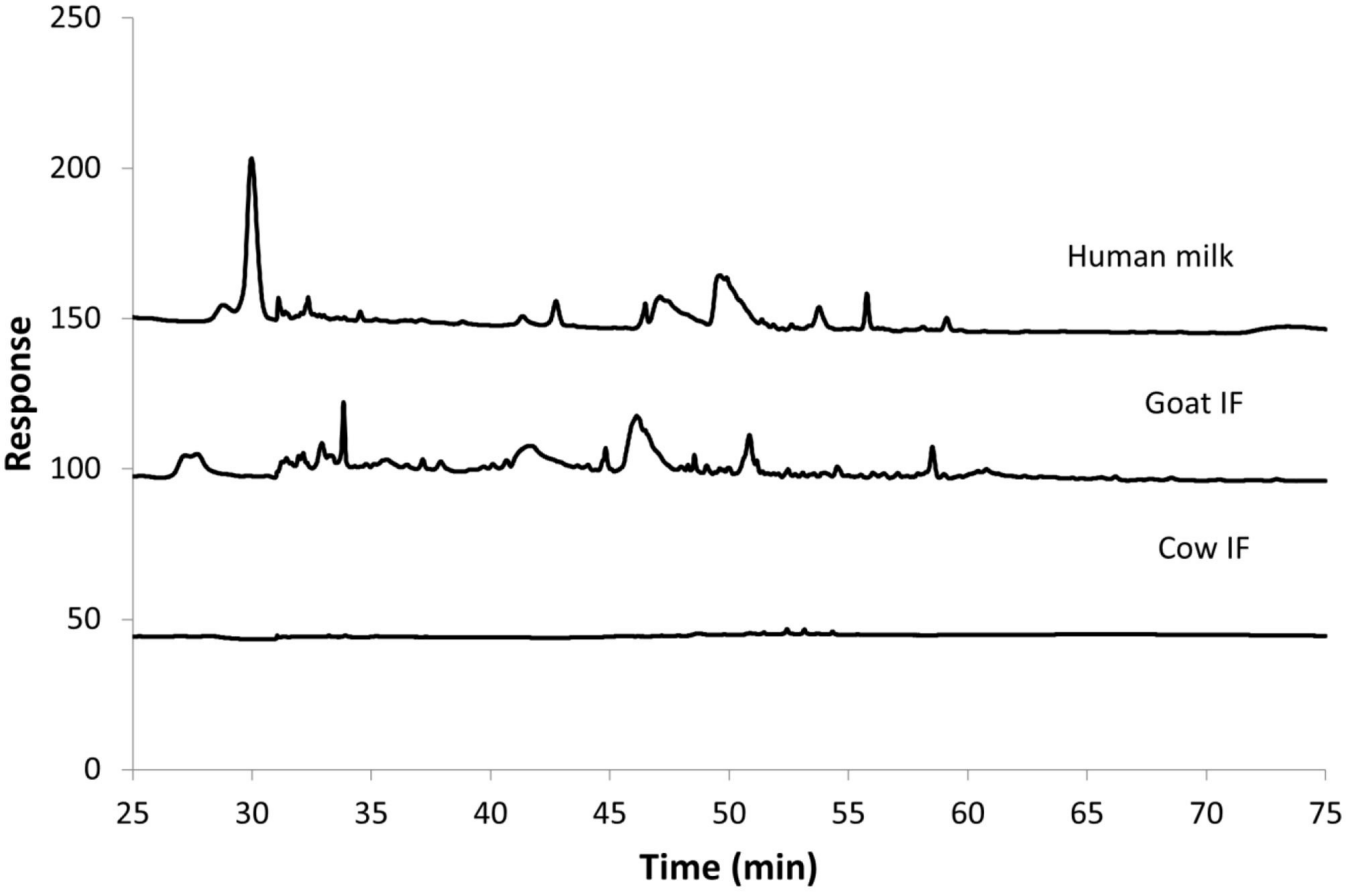
Chromatogram from HPAEC-PAD analysis of Cow IF, Goat IF, and human milk at the end of small intestinal incubation. The oligosaccharide fraction is mainly present from retention time of 30 min.

Figure 3 shows the HPAEC-PAD chromatograms of the different test products at the end of the small intestinal incubation. The oligosaccharides can be seen as peaks from 30 min retention time onwards. Following digestion, human milk contained the most oligosaccharides, followed by goat IF. Cow IF had relatively few oligosaccharides.

### Colonic Incubations—Microbial Metabolic Activity

Gas production was used as a measure for microbial activity. The average (± SD) gas production (kPa) during the first 24 h was 81 ± 6 for goat IF, 75 ± 3 for cow IF, and 85 ± 1 for human milk. All test products resulted in increased gas production compared to the blank incubation (33.9 ± 0.6) (*P* < 0.0001). Human milk and goat IF resulted in more gas production compared to cow IF, but only statistically significantly for human milk (*P* = 0.018) in the first 24 h incubation period. Only a limited and highly variable amount of gas was produced in the second 24 h incubation (9 ± 5 for goat IF, 8 ± 1 for cow IF, 10 ± 3 for human milk, and 12 ± 0 for the blank incubation).

pH was recorded during colonic fermentation as a measure for the intensity of bacterial metabolism. At the beginning of the incubation period the pH of the medium was 6.6 to 6.8 for all test products and blank. After 24 h, pH decreased significantly (*P* < 0.0001) to 5.9 ± 0.0 for all test products, but only marginally (to pH 6.4 ± 0.0) for the blank incubation. There was no further change in pH during the second 24 h incubation period.

The production of SCFAs (acetate, propionate, and butyrate) was analyzed as markers for microbial carbohydrate metabolism. Lactate, produced by lactic acid bacteria in the gut, can also be rapidly converted to acetate, butyrate and propionate by lactate-utilizing gut bacteria (38). The change in concentrations of lactate and SCFAs from 0 to 24 h and 24 to 48 h is reported in Table 2. All test products resulted in increased lactate production during the 0–24 h interval as compared to the blank incubation. Highest lactate levels were observed upon fermentation of goat IF but it was not significantly different to the levels upon fermentation of the cow IF or human milk. The main contribution to total SCFAs was acetate, which increased during the first 24 h of incubation. During the second 24 h incubation period, concentrations of total SCFAs and acetate did not change. There were smaller increases in propionate concentrations in the first 24 h incubation. While propionate further increased in the second 24 h incubation, levels were <1 mM for all incubations. Butyrate concentrations increased <0.5 mM for all test products and the blank during both the 24 and 48 h incubation periods. However, highest butyrate levels were observed upon fermentation of cow IF in the first 24 h incubation period (*P* < 0.0001).

**Table 2:** Change in concentrations of lactate and SCFAs in the 0–24 h and 24–48 h period of colonic fermentation of the goat infant formula (goat IF), cow infant formula (cow IF), and human milk as compared to the blank control.

**Concentration (mM)**	**Goat IF**		**Cow IF**		**Human milk**		**Blank**	
	**0–24 h**	**24–48 h**	**0–24 h**	**24–48 h**	**0–24 h**	**24–48 h**	**0–24 h**	**24–48 h**
Lactate	8.87 ± 1.56^a^	0.44 ± 0.25^a^	7.70 ± 0.54^a^	0.56 ± 0.26^a^	8.26 ± 0.44^a^	−0.97 ± 0.59^b^	1.00 ± 0.06^b^	−0.21 ± 0.04^a^
Acetate	25.22 ± 2.21^a^	−0.04 ± 1.53^a^	24.61 ± 2.73^a^	−0.55 ± 2.81^a^	26.98 ± 1.00^a^	0.44 ± 2.40^a^	12.39 ± 0.53^b^	2.49 ± 0.40^a^
Propionate	3.64 ± 0.09^b^	0.71 ± 0.11^a^	3.57 ± 0.28^b^	0.66 ± 0.35^a^	4.27 ± 0.32^a^	0.64 ± 0.25^a^	2.53 ± 0.10^c^	0.84 ± 0.11^a^
Butyrate	0.12 ± 0.04^c^	0.10 ± 0.11^a^	0.32 ± 0.05^a^	0.28 ± 0.14^a^	0.00 ± 0.00^d^	0.06 ± 0.06^a^	0.22 ± 0.03^b^	0.15 ± 0.04^a^
Total SCFAs	28.99 ± 2.22^a^	0.87 ± 1.75^a^	29.28 ± 2.24^a^	0.20 ± 2.54^a^	31.25 ± 1.12^a^	1.16 ± 2.64^a^	15.16 ± 0.61^b^	3.55 ± 0.55^a^

*SCFAs, short-chain fatty acids. Values for each metabolite with the same letter are not statistically different*.

Microbial protein metabolism results in the production of BCFAs (isobutyrate, isovalerate, and isocaproate). BCFAs were just above the detection limit in the blank incubation (0.03 ± 0.03 mM) during the first 24 h of incubation, but not in any of the test products. In the second 24 h, concentrations of branched SCFAs were 0.07 ± 0.04 mM from the incubations with blank and the cow IF. Both goat IF and human milk had no detectable change in BCFAs.

The production of ammonium (${\text{NH}}_{4}^{+}$) that results from proteolytic activity of the gut microbiota was significantly reduced (*P* = 0.0316) by all products compared to the blank incubation. The highest ammonium production occurred during the 0–24 h time interval (59 ± 5 mg/L for goat IF, 50 ± 8 mg/L for cow IF, 57 ± 4 mg/L for human milk, and 87 ± 2 mg/L for the blank). During the second 24 h incubation period, ammonium concentrations were 24 ± 1 mg/L for goat IF, 28 ± 2 mg/L for cow IF, 35 ± 22 mg/L for human milk, and 39 ± 6 mg/L for the blank.

### Colonic Incubations—Microbial Composition

**Figure 4:**
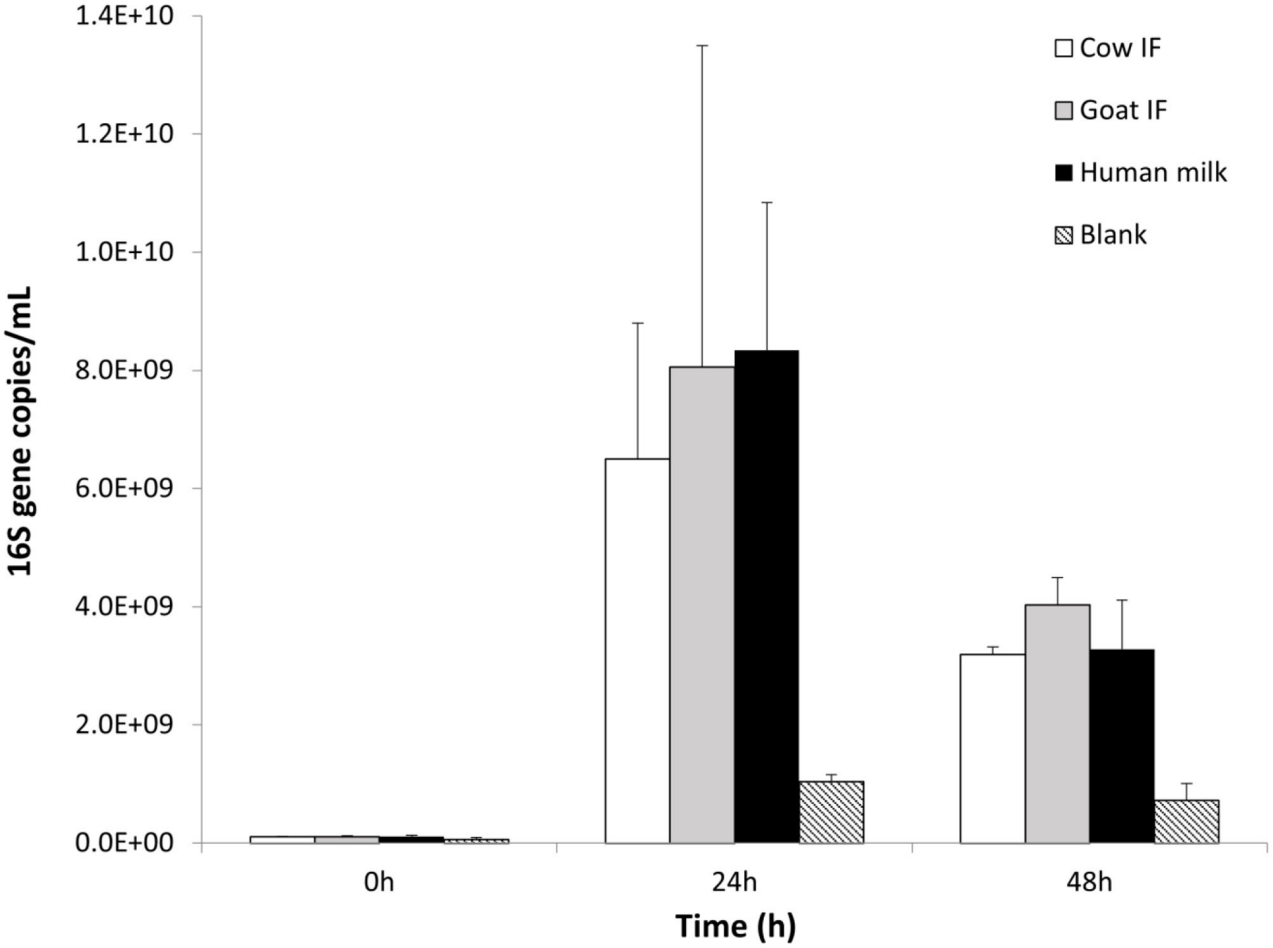
*Bifidobacterium* levels (16S copies/mL) at 0, 24, and 48 h of colonic fermentation of the three test products as compared to the blank control.

The original donor sample selected for the *in vitro* dynamic digestive model was dominated by the *Bifidobacteriaceae* family, and in particular *Bifidobacteriaceae* operational taxonomic unit (OTU) (related to *Bifidobacterium Breve*). All test products resulted in a further increase in numbers of *Bifidobacteriaceae* as compared to the blank incubation (*P* = 0.0001), with the main increase being observed during the first 24 h of incubation (Figure 4). No differences were observed amongst the different test products.

At the start of the incubation, the bacterial inoculum was mainly dominated by bacterial species of the *Bifidobacteriaceae* (Table 3). *Lachnospiraceae* was the next most abundant bacterial species. *Lactobacillus* was below the limit of detection. Reciprocal Simpson Diversity index indices for goat IF, cow IF and human milk were 2.9, 2.8, and 2.9, respectively, compared to the blank incubation with an index of 6.4. These reflect a strong increase in abundance of *Bifidobacteriaceae* and *Enterobacteriaceae*, following the incubation with the test products (Table 3).

**Table 3:** Average (*n* = 3) abundance (%) of dominant bacterial families in the original inoculum and following 48 h incubation with digestive products of goat infant formula (goat IF), cow infant formula (cow IF), and human milk and with the blank.

**Phylum**	**Family**	**Inoculum**	**Goat IF**	**Cow IF**	**Human milk**	**Blank**
*Actinobacteria*	*Bifidobacteriaceae*	55.3	44.8	48.7	36.7	11.0
	*Eggerthellaceae*	0.5	0	0	0	2.0
*Bacteroidetes*	*Bacteroidaceae*	9.5	7.5	7.0	7.1	24.6
	*Rikenellaceae*	0.1	0	0	0	0
*Firmicutes*	*Acidaminococcaceae*	2.8	4.8	3.6	6.3	1.5
	*Clostridiaceae*	0.2	0.1	0.3	0.1	0
	*Enterococcaceae*	1.1	1.7	1.7	1.3	22.8
	*Erysipelotrichaceae*	5.0	0	0	0	0
	*Lachnospiraceae*	18.4	2.7	3.5	1.9	20.0
	*Ruminococcaceae*	1.0	0	0	0	0.4
	*Streptococcaceae*	0.3	0	0	0	0
*Proteobacteria*	*Burkholderiaceae*	0.5	0	0	0	0.1
	*Enterobacteriaceae*	5.3	38.4	34.9	46.5	17.4

## Discussion

Human, goat and cow milk contain natural prebiotics such as oligosaccharides, lactose, nucleotides, and glycosylated proteins and lipids, albeit at different concentrations and diversity (15). HMOs are the third most abundant components of human milk and therefore have a greater prebiotic effect than other components (6).

All test products stimulated overall microbial activity as observed by a stronger pH decrease, increased gas production and production of health-related metabolites such as SCFAs and lactate as compared to the blank incubation. Gas production reflects microbial substrate fermentation while the decrease in pH was likely due to increased lactate and SCFAs. These observations are consistent with the effects of human milk on microbial activity in humans (3, 8) and studies on goat and cow milk in rodents (20, 21).

With respect to product-specific findings, it was found that human milk and goat IF digestion and fermentation resulted in a significantly greater gas production compared to cow IF, while human milk digestion, and fermentation resulted in a slightly greater increase in acetate and propionate production compared to either of the formulas. While both formulas resulted in increased lactate production compared to the blank incubation, highest lactate levels were observed upon fermentation of goat IF. Lactate is an important metabolite in the human colon environment because it decreases the gut pH and acts as an antimicrobial agent (39), but also because it is the driver of a series of trophic interactions with other bacteria, resulting in the production of butyric and propionic acids (40). Thus, changes in lactate could have resulted either from a change in production or utilization. However, concentrations of propionate were similar between the two formulas and while butyrate was higher with cow IF, butyrate represented only 1–2% of the total output of SCFAs.

With regards to bacterial composition, all test products increased *Bifidobacteriaceae* and reduced species richness compared to the blank during the incubation period. The major change in bacterial composition was increased abundance of *Enterobacteriaceae*, mainly attributed to *Enterobacteriaceae* OTU (related to *Escherichia coli*). These changes are consistent with the production of SCFAs and lactate observed upon treatment with all test products. For example, *Escherichia coli* utilizes a wide variety of substrates including HMOs (41, 42) to produce acetate. Both *Bifidobacteriaceae* and *Enterobacteriaceae* are capable of producing high concentrations of lactate. *Acidaminococcaceae* also increased, mainly due to *Acidaminococcaceae* OTU (related to *Phascolarctobacterium faecium*), which is a known producer of propionate. While the abundance of *Bifidobacteriaceae* and *Enterobacteriaceae*, it is likely that levels of some bacterial species remained unchanged, which was not possible to demonstrate using proportional 16S sequencing but could be in the future using quantitative sequencing (43).

Lactate-producing bacteria are primary colonizers of the infant gut (44). Therefore, to prevent toxic accumulation, lactate must be used as a substrate by lactate-utilizing bacteria. This may result in H_2_ production and accumulation, which may contribute to infantile colic symptoms such as acute bloating and cramping (44). A small study (44) showed that an imbalance between H_2_-producing and H_2_-utilizing bacteria was associated with infantile colic. In the present study, fermentation of goat IF resulted in higher lactate levels, albeit not significantly, and gas production was highest during fermentation of goat IF and human milk. It would be of interest to explore these results in future clinical settings.

In the present system, *Lactobacilli* levels remained below the detection limit, consistent with low levels during infancy reported in other studies (3, 24). After *Bifidobacteriaceae, Lachnospiraceae* was the next most abundant bacterial species in the inoculum. Tannock et al. (24) observed that when *Bifidobacteriaceae* abundance in stools of infants was high, *Lachnospiraceae* abundances tended to be low, suggesting there is a metabolically competitive interaction between *Bifidobacteriaceae* and *Lachnospiraceae*. In the present study, the percentage of *Lachnospiraceae* dropped after incubation with digestive products of formulas and human milk, suggesting that the digestive products from human milk and formula were more conducive to growth of *Bifidobacteriaceae* than *Lachnospiraceae*. Thus, similar to Tannock et al. (24), the present study shows that *Bifidobacteria* are maintained even in the presence of low levels of oligosaccharides in formula made with ruminant milks. In the present study, both formulas contained milk fat, but as the goat IF was made with whole goat milk without added whey, it contained higher levels of milk fat than the cow IF (50 vs. 30% of total lipids). As a result, the goat IF would be expected to contain more components of the milk fat globule membrane (MFGM). It is possible that glycoproteins and glycolipids associated with the MFGM may also act as growth substrates for *Bifidobacteria* and other bacteria (45–47). To note as well, that the cow IF in the present study contained more milk fat than standard infant formulas manufactured with skim milk and whey protein ingredients, which may have contributed to some effects on microbial activity and composition (46). In addition, both formulas contained DHA, which may play a role in the development of the microbiota and allergy in infants (48).

One of the limitations of the *in vitro* dynamic digestive model is that it lacks the full complement of the digestive system. For example, brush border enzymes, such as lactase, are not present and as a result there was no breakdown of lactose into galactose or glucose. All test products were diluted by half and then a 3.5 kDa dialysis membrane was used during the jejunal and ileal incubation phase to simulate the absorptive processes and to reduce undigested lactose passing into the colonic digestion phase. Dialysis reduced lactose concentrations by half what was present in the diluted test products. HPAEC-PAD analysis confirmed there was no loss of oligosaccharides after dialysis, which is important when performing mechanistic research on the effect of the test products on the colonic microbiota. The highest amount of non-digestible oligosaccharides at the end of the small intestinal incubation was present in human milk as compared to the goat or cow IF, consistent with much higher concentrations of oligosaccharides from human milk compared to ruminant milks (19). Even with the dialysis, it is possible that concentrations of lactose and amino-nitrogen presented to the bacteria within the inoculum may be higher than levels *in vivo*. However, levels of BCFAs that are indicative of protein fermentation (49) were only just detectable in the second 24 h of incubation with blank or cow IF when it may be expected that supply of fermentable carbohydrates becomes limiting. Thus, it is likely that protein digestion products at least had little impact on the outcomes of this study. While infants are able to digest large quantities of lactose, it can be expected that some lactose escapes digestion and absorption and is fermented by gut bacteria (50, 51).

The strength of the *in vitro* dynamic digestive model is the tight control of environmental factors influencing the microbiota. Thus, it is possible to be very confident that the stimulation of specific bacterial species is directly attributed to the inherent prebiotic properties of the products and not to other events. This, combined with the ability to test products in triplicate with the single inoculum, provides much greater reproducibility than using *in vivo* studies with infants.

## Conclusions

All three milks stimulated microbial activity and increased *Bifidobacteria*, which are regarded as beneficial saccharolytic bacteria in infancy (3). Similar to Tannock et al. (24), both formulas impacted the gut microbial activity and community composition comparable to human milk, despite the relative absence of oligosaccharides in the formulas. This may be explained by the presence of naturally-occurring oligosaccharides, milk fat and MFGM within the formulas, in particular the formula made from whole goat milk, used in the present study. Further clinical research is warranted on the role of goat milk fat in formulas on the development of the gut microbiota in early life.

## Data Availability Statement

The datasets presented in this study can be found in online repositories. The names of the repository/repositories and accession number(s) can be found at: NCBI; accession no. PRJNA675453.

## Ethics Statement

The studies involving human participants were reviewed and approved by Ethics Committee of Ghent University Hospital. Written informed consent to participate in this study was provided by the participant or the participants' legal guardian/next of kin.

## Author Contributions

CP developed the study concept. PV carried out the experiment and collected the data. SG, CP, and PV evaluated the data, wrote the manuscript, and proofread and approved the final manuscript. All authors contributed to the article and approved the submitted version.

## Conflict of Interest

SG and CP are employees of Dairy Goat Co-operative (NZ) Ltd, that financially supported the study and manufactured the formulas for use in this study. The remaining author declares that the research was conducted in the absence of any commercial or financial relationships that could be construed as a potential conflict of interest.

## Abbreviations

BCFA: branched-chain fatty acids
GIT: gastrointestinal tract
HMO: human milk oligosaccharide
HPAEC-PAD: high-pressure anion-exchange chromatography with pulsed amperometric detection
IF: infant formula
MFGM: milk fat globule membrane
SCFA: short-chain fatty acid:
SHIME: simulator of the human intestinal microbial ecosystem.
